# From Papillary Muscle Rupture to Left Atrial Pseudoaneurysm: A Rare Sequential Complication After Acute Myocardial Infarction and Mitral Valve Replacement

**DOI:** 10.7759/cureus.114274

**Published:** 2026-08-10

**Authors:** Rachelle Makhoul, Laurence Barde, Noureddine El Hajjaji, Nassim Sekkal, Jaweher Arfaoui

**Affiliations:** 1 Cardiology, Centre Hospitalier de Montauban, Montauban, FRA; 2 Cardiology, Centre Hospitalier Universitaire de Toulouse, Toulouse, FRA

**Keywords:** acute mitral regurgitation, acute myocardial infarction, left atrial pseudoaneurysm, multimodality imaging, papillary muscle rupture

## Abstract

Papillary muscle rupture is a rare but life-threatening mechanical complication of acute myocardial infarction, resulting in acute severe mitral regurgitation and cardiogenic shock. Emergency surgical intervention is essential for survival. Left atrial intramural hematoma and subsequent pseudoaneurysm formation are exceptionally rare complications of mitral valve surgery, with no established management strategy.

We report the case of a 77-year-old man presenting with acute severe mitral regurgitation secondary to complete rupture of the posteromedial papillary muscle following an inferior myocardial infarction. He underwent emergency mitral valve replacement with a favorable postoperative course. Routine postoperative imaging unexpectedly revealed a left atrial wall hematoma, which evolved into a stable left atrial pseudoaneurysm on follow-up cardiac computed tomography. Given the absence of symptoms or lesion progression, conservative management with imaging surveillance was adopted. This case highlights the value of multimodality imaging in the diagnosis and follow-up of rare postoperative complications and supports a conservative approach in carefully selected clinically stable patients.

## Introduction

Mechanical complications of acute myocardial infarction (AMI) have become uncommon in the contemporary era owing to widespread access to early coronary reperfusion strategies [1]. Nevertheless, they remain among the most life-threatening consequences of myocardial infarction and are associated with substantial morbidity and mortality [2]. Papillary muscle rupture is a rare but catastrophic mechanical complication that typically occurs within the first week following an infarction and results in acute severe mitral regurgitation, rapidly leading to pulmonary edema and cardiogenic shock if left untreated [3]. Prompt diagnosis by echocardiography and emergency surgical intervention are essential to improve survival [4].

Transthoracic and transesophageal echocardiography are the imaging modalities of choice for establishing the diagnosis, defining the mechanism of mitral regurgitation, and guiding surgical planning [4]. Urgent surgical intervention remains the standard of care for papillary muscle rupture [5]. Although the choice between mitral valve repair and replacement remains controversial, repair is generally favored when feasible, whereas replacement is often required in cases of extensive papillary muscle necrosis [5-6].

Left atrial intramural hematoma is an exceptionally rare complication, most commonly reported after mitral valve surgery, but also described following catheter-based interventions, other cardiac procedures, and trauma [7]. Progression to a left atrial pseudoaneurysm is even less frequently reported, and its optimal management remains uncertain because available evidence is limited to isolated case reports and small case series [8-9]. Management ranges from urgent surgical intervention in hemodynamically unstable patients to conservative imaging surveillance in carefully selected stable individuals [8-9].

We present a rare case of complete posteromedial papillary muscle rupture complicating an inferior myocardial infarction, successfully treated by emergency mitral valve replacement, followed by the unexpected development of a persistent left atrial pseudoaneurysm secondary to presumed coronary sinus injury, which was managed conservatively with a favorable short-term outcome.

## Case presentation

A 77-year-old man with a history of hypertension and epilepsy, treated with hydrochlorothiazide (Esidrex®), ramipril, and phenobarbital (Gardenal®), was admitted to the emergency department for acute respiratory failure. Three days prior to admission, he had experienced chest pain that was not investigated at the time. On admission, the patient presented with acute pulmonary edema and severe respiratory distress despite initial optimal medical therapy, including non-invasive ventilation and intravenous furosemide. Electrocardiography revealed pathological Q waves in the inferior leads, consistent with a late-presenting inferior myocardial infarction. Laboratory investigations showed elevated cardiac troponin and B-type natriuretic peptide levels, associated with an inflammatory biological syndrome (Table 1). Renal function and serum lactate levels were normal.

**Table 1:** Laboratory data

Parameters	Patient Values	Reference Range
Complete Blood Count		
White blood cell count	18 ×10^9^/L	4.0–11.0 ×10^9^/L
Hemoglobin	14 g/dl	13.5–17.5 g/dL
Hematocrit	40%	41–53%
			Red blood cell count	4.22 ×10^12^/L	4.5–5.9 ×10^12^/L
Mean corpuscular volume	94 fL	80–100 fL			
			Platelet count	321 ×10^9^/L	150–400 ×10^9^/L
Neutrophils	16.94 ×10^9^/L	1.85-8.14 ×10^9^/L			
			Lymphocytes	0.77 ×10^9^/L	1.24-3.62 ×10^9^/L
Monocytes	0.97 ×10^9^/L	0.23-0.72 ×10^9^/L			
			Eosinophils	0.00 ×10^9^/L	0.05-0.58 ×10^9^/L
Basophils	0.04 ×10^9^/L	0.00-0.09 ×10^9^/L			
			Biochemistry Panel		
Sodium	132 mmol/L	135–145 mmol/L
			Potassium	4.6 mmol/L	3.5–5.1 mmol/L
Chloride	96 mmol/L	98–107 mmol/L			
Bicarbonate	22 mmol/L	22–29 mmol/L
Creatinine	108 µmol/L	59-104 µmol/L
ALT	24 U/L	0-41 U/L
			AST	25 U/L	<40 U/L
GGT	113 U/L	8-61 U/L			
			Bilirubin (Total)	1 mg/dL	0.1–1.2 mg/dL
Cardiac Markers					
			Pro-BNP	4539 ng/L	<125 ng/L
Troponin H0	457 ng/L	<14 ng/L
			Troponin H1	482 ng/L	<14 ng/L
Inflammatory Markers					
			C-reactive protein	161 mg/L	<5 mg/L
Coagulation Markers					
TP	88%	70-100%
			Fibrinogen	8.18 g/L	2.38-5.00 g/L
D-dimer	2210 ng/ml	<500 ng/mL

Transthoracic echocardiography (TTE) demonstrated acute severe mitral regurgitation caused by rupture of the subvalvular apparatus, with findings suggestive of papillary muscle rupture. Left ventricular systolic function was preserved (left ventricular ejection fraction (LVEF) 70%), with a non-dilated left ventricle and left atrium. Right ventricular function was also preserved (Video 1).

**Video 1:** Echocardiographic evidence of papillary muscle rupture and acute mitral regurgitation

The patient was urgently transferred to our tertiary referral center for surgical management and was taken directly to the operating room. Coronary angiography demonstrated occlusion of the posterior interventricular artery (Video 2). Intraoperative transesophageal echocardiography (TEE) confirmed massive acute mitral regurgitation secondary to complete rupture of the posteromedial papillary muscle (Video 3). 

**Video 2:** Coronary angiography showing occlusion of the posterior interventricular artery

**Video 3:** Massive mitral regurgitation with complete rupture of the posteromedial papillary muscle on TEE TEE: Transesophageal Echocardiography

Emergency mitral valve replacement was performed using a 31-mm Epic™ bioprosthetic valve. The immediate postoperative course was favorable, with rapid discontinuation of inotropic and vasopressor support, followed by early extubation. Therapeutic anticoagulation with unfractionated heparin was initiated, along with continued diuretic therapy for persistent signs of congestion.

**Figure 1:**
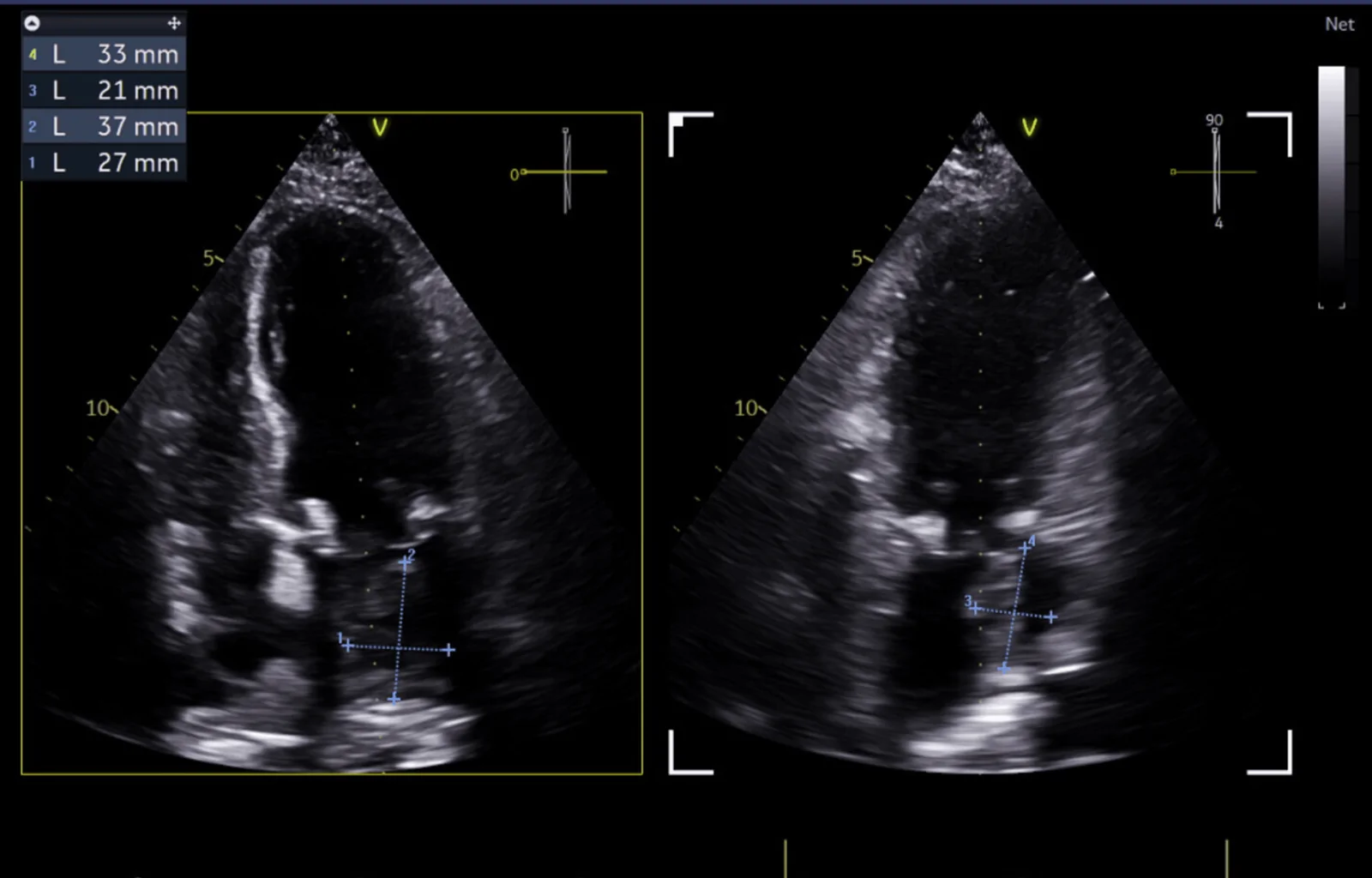
Postoperative transthoracic echocardiography (TTE) images of the patient Apical four-chamber (left-hand side image) and two-chamber (right-hand side image) views, demonstrating a left atrial hematoma along the posterior wall of the left atrium.

Postoperative transthoracic echocardiography showed a non-dilated left ventricle with moderately reduced systolic function (LVEF 45%) and homogeneous regional wall motion. The mitral bioprosthesis was well positioned and functioning normally, with a mean transvalvular gradient of 7 mmHg, a Doppler velocity index of 1.8, and no evidence of paravalvular or transvalvular regurgitation. Unexpectedly, a 4 × 1.5 cm hematoma (Video 4, Figure 1) was identified along the posterior wall of the left atrium, adjacent to the coronary sinus, raising suspicion of a possible intraoperative coronary sinus injury. The remainder of the examination was unremarkable. Cardiac computed tomography confirmed the presence of the left atrial wall hematoma. 

**Video 4:** Hematoma along the posterior wall of the left atrium post mitral valve replacement on TTE TTE: Transthoracic Echocardiography

**Figure 2:**
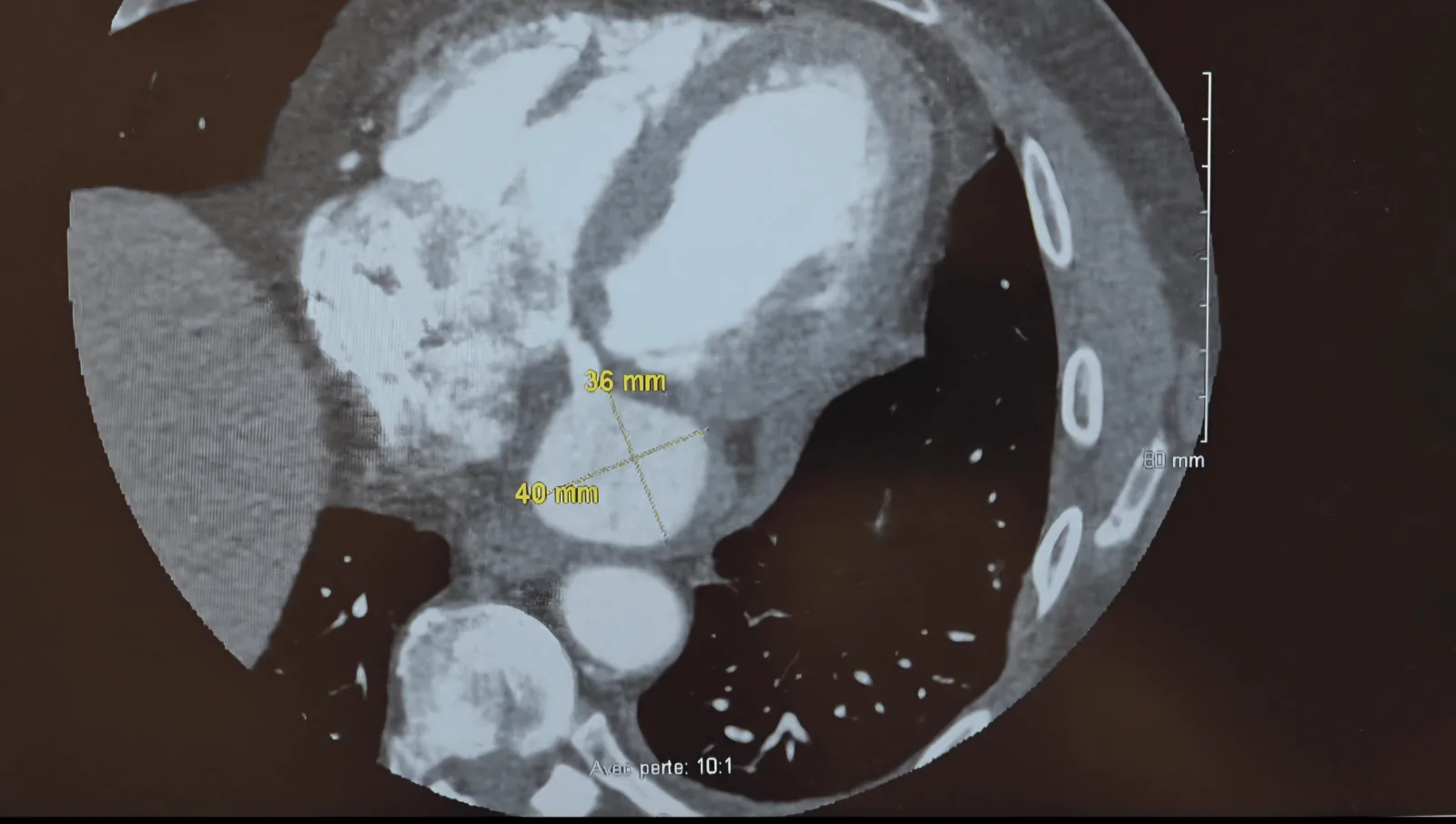
Contrast-enhanced cardiac computed tomography (CECT) performed three months after mitral valve replacement The image is demonstrating a persistent, stable left atrial pseudoaneurysm measuring 40 × 36 mm adjacent to the posterior wall of the left atrium.

Serial transthoracic echocardiography performed during the following days demonstrated recovery of left ventricular systolic function (LVEF 57%), a normally functioning mitral bioprosthesis without obstruction, and no increase in the size of the left atrial hematoma. Given the patient's clinical stability and the absence of imaging progression, conservative management was pursued, and the patient was discharged with close follow-up. Because of the significant interaction between vitamin K antagonists and phenobarbital, long-term therapeutic anticoagulation was maintained with low-molecular-weight heparin for three months. Cardiac computed tomography performed three months after surgery demonstrated persistence of a stable 40 × 35 mm pseudoaneurysm arising from the posterior wall of the left atrium (Video 5, Figure 2). In the absence of symptoms, lesion enlargement, or established therapeutic guidelines, a conservative management strategy with planned CT surveillance at six months was adopted.

**Video 5:** Pseudoaneurysm arising from the posterior wall of the left atrium on cardiac CT CT: Computed tomography

## Discussion

Papillary muscle rupture (PMR) is a rare but catastrophic mechanical complication of acute myocardial infarction (AMI), occurring in less than 1% of patients in the contemporary reperfusion era [1]. It most frequently involves the posteromedial papillary muscle because of its single blood supply from the posterior descending artery, explaining its association with inferior myocardial infarction [2], as observed in our patient. Without prompt surgical intervention, PMR rapidly leads to acute severe mitral regurgitation, pulmonary edema, and cardiogenic shock, with a very high mortality rate [3]. Early diagnosis by echocardiography and emergency mitral valve surgery remain the standard of care [4]. 

The choice between mitral valve repair and replacement depends on the underlying pathology and the feasibility of achieving a durable repair. Mitral valve repair is recommended whenever technically feasible for degenerative mitral regurgitation because it preserves left ventricular function and is associated with improved long-term survival [4]. However, in the setting of acute ischemic papillary muscle rupture, valve replacement is performed more frequently because extensive papillary muscle necrosis and leaflet tethering often preclude a durable repair. Mitral valve repair may be considered in selected patients with partial papillary muscle rupture, limited tissue destruction, and favorable valve anatomy, particularly in experienced centers, whereas complete papillary muscle rupture or extensive structural damage generally necessitates mitral valve replacement to ensure procedural durability and hemodynamic stability [4].

The postoperative finding of a left atrial intramural hematoma, which subsequently evolved into a stable pseudoaneurysm, represents the unique aspect of this case. Left atrial intramural hematoma is an exceptionally uncommon complication, most often reported after mitral valve surgery, which accounts for 56.3% of published cases (47.1% after mitral valve replacement and 9.2% after mitral valve repair) [7]. Injury to the atrioventricular groove or coronary sinus has been proposed as one of the underlying mechanisms, particularly when the lesion is located along the posterior left atrial wall [8], as in our patient.

Because of its rarity, the optimal management of left atrial intramural hematoma and pseudoaneurysm remains uncertain [7]. Published cases suggest that surgical intervention is indicated in patients with hemodynamic instability, prosthetic valve dysfunction, progressive expansion, or obstruction of left atrial inflow [8], whereas conservative treatment with close imaging follow-up has been successfully adopted in clinically stable patients.

In our case, serial transthoracic echocardiography and cardiac computed tomography demonstrated stability of the lesion without prosthetic dysfunction or clinical deterioration, supporting a conservative approach. This management strategy is consistent with previously reported cases in which careful imaging surveillance resulted in favorable outcomes.

Finally, this case highlights the complementary role of multimodality imaging throughout the patient's course. Transthoracic and transesophageal echocardiography were essential for diagnosing papillary muscle rupture and assessing prosthetic valve function [4], whereas cardiac computed tomography accurately characterized the postoperative lesion and enabled non-invasive follow-up. This multimodality approach facilitated appropriate therapeutic decision-making and avoided unnecessary reintervention in a clinically stable patient.

## Conclusions

This case highlights the complexity of managing rare mechanical complications of acute myocardial infarction and their unexpected postoperative sequelae. While emergency mitral valve replacement remains the cornerstone of treatment for ischemic papillary muscle rupture, postoperative left atrial pseudoaneurysm poses a challenging therapeutic dilemma because of its rarity and the lack of consensus recommendations. Our experience emphasizes the value of multimodality imaging for diagnosis and longitudinal assessment and suggests that, in the absence of clinical or imaging evidence of progression, a conservative strategy with careful surveillance may be a feasible and effective management option.
